# Primary Care Physicians’ Experience Using Advanced Electronic Medical Record Features to Support Chronic Disease Prevention and Management: Qualitative Study

**DOI:** 10.2196/13318

**Published:** 2019-11-29

**Authors:** Rana Melissa Rahal, Jay Mercer, Craig Kuziemsky, Sanni Yaya

**Affiliations:** 1 Population Health Program University of Ottawa Ottawa, ON Canada; 2 Bruyère Continuing Care Ottawa, ON Canada; 3 Office of Research Services MacEwan University Edmonton, AB Canada; 4 School of International Development and Global Studies University of Ottawa Ottawa, ON Canada; 5 The George Institute for Global Health University of Oxford Oxford United Kingdom

**Keywords:** electronic health record, chronic disease, primary health care, medical informatics

## Abstract

**Background:**

Chronic diseases are the leading cause of death worldwide. In Canada, more than half of all health care spending is used for managing chronic diseases. Although studies have shown that the use of advanced features of electronic medical record (EMR) systems improves the quality of chronic disease prevention and management (CDPM), a 2012 international survey found that Canadian physicians were the least likely to use 2 or more EMR system functions. Some studies show that maturity vis-à-vis clinicians’ EMR use is an important factor when evaluating the use of advanced features of health information systems. The Clinical Adoption Framework (CAF), a common evaluation framework used to assess the success of EMR adoption, does not incorporate the process of maturing. Nevertheless, the CAF and studies that discuss the barriers to and facilitators of the adoption of EMR systems can be the basis for exploring the use of advanced EMR features.

**Objective:**

This study aimed to explore the factors that primary care physicians in Ontario identified as influencing their use of advanced EMR features to support CDPM and to extend the CAF to include primary care physicians’ perceptions of how their use of EMRs for performing clinical tasks has matured.

**Methods:**

Guided by the CAF, directed content analysis was used to explore the barriers and facilitating factors encountered by primary care physicians when using EMR features. Participants were primary care physicians in Ontario, Canada, who use EMRs. Data were coded using categories from the CAF.

**Results:**

A total of 9 face-to-face interviews were conducted from January 2017 to July 2017. Dimensions from the CAF emerged from the data, and one new dimension was derived: physicians’ perception of their maturity of EMR use. Primary care physicians identified the following key factors that impacted their use of advanced EMR features: performance of EMR features, information quality of EMR features, training and technical support, user satisfaction, provider’s productivity, personal characteristics and roles, cost benefits of EMR features, EMR systems infrastructure, funding, and government leadership.

**Conclusions:**

The CAF was extended to include physicians’ perceptions of how their use of EMR systems had matured. Most participants agreed that their use of EMR systems for performing clinical tasks had evolved since their adoption of the system and that certain system features facilitated their care for patients with chronic diseases. However, several barriers were identified and should be addressed to further enhance primary care physicians’ use of advanced EMR features to support CDPM.

## Introduction

### Background

According to the World Health Organization, by 2020, chronic diseases will account for 73% of all deaths and 60% of the global burden of disease [1]. The World Health Organization recommends that chronic disease prevention must focus on controlling risk factors such as high blood pressure and tobacco use [1].

Electronic medical records (EMRs) are one of many initiatives available in high-income countries to assist in addressing these risk factors. In a systematic review, approximately 67% of studies showed that EMRs have a positive effect on preventive care, and about 57% of studies found that EMRs contribute to a modest improvement in disease management [2].

Electronic reminder features for preventive or follow-up care automate reminders for specific tests (eg, vaccinations and blood tests) based on recommended guidelines [3]. Advanced EMR features, such as electronic reminders, have been shown to support chronic disease prevention and management (CDPM). When EMR reminders were combined with access to EMR information (eg, history of hypertension and cardiovascular disease), 28% of the patient population was found to be at risk for undiagnosed type 2 diabetes [4].

A grounded theory study of EMR usage ranked EMR features from basic to advanced [5]. Advanced features included automated reminders for tests and screening; using decision support tools, such as a cardiovascular risk tool; using a recall system to search for patients with a specific condition; creating customized templates, such as diabetic flow sheets; and using a graph feature to view the trend of a patient’s test results over time [5].

### Statement of the Problem

Not all physicians use the advanced features of EMR systems to support CDPM. A 2012 study showed that Canadian physicians were the least likely to use at least two EMR functions [6]. Thus, there is a gap in our understanding of the barriers to and facilitating factors of the use of advanced features in EMR systems.

### Factors That Impact the Adoption of Electronic Medical Records

Much of the literature has focused on the factors that contribute to successful EMR adoption. Studies have discussed the need for EMR champions and staff participation to encourage adoption [7-9]. Rogers’ diffusion of innovations theory suggests that the characteristics of potential adopters are also a key factor for EMR adoption [10].

In addition, studies have identified the importance of providing adequate education and training to support EMR adoption [11,12]. In the Canadian province of Ontario, the Association of Family Health Teams developed a program comprising individuals known as quality improvement decision support specialists (QIDSS) who were available on-site to assist teams to access and better use EMR data to improve care [13].

Furthermore, some studies have highlighted the importance of advancing the level of health information system (HIS) use to obtain improved clinical outcomes and have suggested that benefits grow over time as users gain experience, as improvements are made in systems, and as workflows are adjusted to users’ needs [14,15]. A Canadian study in Ontario assessed the progress in the use of advanced EMR features and found a direct correlation between years of EMR use and EMR maturity [14]. Thus, in evaluating the use of advanced features of EMR systems, it is important to consider how the use of EMR systems by clinicians has evolved since EMR adoption.

### Conceptual Framework

In this study, the Clinical Adoption Framework (CAF) [16] was used to categorize the study results and to explore the barriers and facilitators that primary care physicians encounter when using EMR features to support CDPM. Although the CAF does not evaluate the maturity of a clinician’s HIS use, the framework is appropriate for this study as it identifies microlevel, mesolevel, and macrolevel factors that influence EMR success.

Several frameworks for HIS adoption have been reported in the literature [16-21]. OntarioMD, a cooperative owned by the Ontario Medical Association and funded by the provincial government, is responsible for certifying EMRs in Ontario [22]. OntarioMD developed the EMR Maturity Model [21] to help clinicians optimize their EMR use by evaluating their level of EMR use. The model evaluates maturity in terms of how the product is used, and users can measure their maturity level for a certain function (eg, appointment scheduling and laboratory results) across 6 maturity levels (see Multimedia Appendix 1) [21]. Thus, this study refers to maturity as the maturity of the user’s skill set and clinical processes in using the HIS, rather than the maturity of a product (ie, type of features implemented). The EMR Maturity Model is based on existing models such as the CAF.

The CAF (shown in Multimedia Appendix 2) proposes that successful clinical adoption of HISs at the microlevel depends on the following dimensions: the quality of the system’s performance, information, and support service provided for the HIS; its use and user satisfaction; and net benefits. At the mesolevel, the people involved, the organization, and the implementation of the HIS have a direct effect on the microlevel HIS adoption by health care professionals. At the macrolevel, successful clinical adoption depends on health care standards; funding and incentives; legislation/policy and governance; and societal, political, and economic trends. A detailed description of the dimensions for each level can be found in previous studies [16,17,23].

### Purpose of the Study

This study explored the barriers primary care physicians encounter while using advanced EMR features to facilitate CDPM and the factors facilitating their use of these features. Furthermore, this study extends the CAF to include primary care physicians’ perceptions of how their use of the EMR system had evolved. Thus, the main contribution of this study was looking at the CAF and the maturity of EMR use from the perspective of primary care providers, as they are the ones managing chronic illness.

## Methods

### Study Setting and Design

On the basis of existing evidence about factors influencing EMR adoption, a qualitative directed content analysis was conducted using the CAF. A directed content analysis is typically used when existing theory or prior research about a phenomenon needs further description to validate or extend a theoretical framework or theory [24]. Thus, we used directed content analysis to extend the CAF.

The study was conducted at primary care clinics located in the Canadian province of Ontario. Although there are various EMR systems available in Ontario, the most common systems used at primary care clinics are PS Suite EMR (produced by Telus Health) [25], Nightingale On Demand (produced by Telus Health) [26], IndiviCare (produced by Indivica) [27], and OSCAR (produced by OSCAR EMR Inc) [28]. Advanced EMR features available in these systems include but are not limited to the following:

- Drug databases that provide dosing information, administration, and medication allergy alerts.
- Hospital Report Manager [29], an Ontario provincial feature used to electronically integrate patient reports (eg, medical records and diagnostic imaging reports) from hospitals and specialty clinics directly into a patient’s chart.
- Ontario Laboratories Information System (OLIS) that automatically receives laboratory results from hospitals directly into the patient’s chart [30].
- Electronic fax to electronically receive faxed documents into EMRs.

### Study Participants, Sampling, and Recruitment

Eligible participants were primary care physicians located in Ontario who had used EMRs for at least one year. Purposeful sampling was used to represent a range of ages (less than 30 years, 30-40 years, 41-50 years, 51-60 years, 61-70 years, and greater than 71 years), sexes (female and male), and individuals from different cities in Ontario. Face-to-face interviews were conducted.

Data saturation determined the sample size. After 7 interviews, no new ideas were being introduced. Nevertheless, 2 more interviews were conducted to validate that saturation had occurred. A similar study exploring primary care physicians’ experience with EMRs also had a sample size of 9 participants [31].

OntarioMD assisted in recruiting participants by sharing an advertisement about this study with its peer leaders. Similarly, Ontario academic family practices were contacted to identify participants, resulting in the Ottawa Hospital Family Health Team reaching out to its members. Recruitment emails were also sent to individual family practices.

### Data Collection and Research Instruments

Data were actively collected between January 2017 and July 2017 by the primary author (RR). In-person interviews were audio recorded. Interviews were approximately 20 min to 60 min and were conducted by using a semistructured interview guide (Multimedia Appendix 3). The interview guide was pilot-tested in July 2016 with a primary care physician.

### Data Analysis

Audio recordings of interviews were transcribed verbatim. The directed content approach using the CAF helped determine the initial coding scheme [16,17]. Each interview transcript was read line by line; any text that appeared to describe a barrier or facilitating factor was highlighted (RR). Next, NVivo software (QSR International) [32] was used to help code all highlighted text using predetermined codes (RR). Data that could not be coded into one of the categories of the CAF were coded with a label that captured the essence of the barrier or facilitating factor. Finally, 2 team members (RR and SY) independently analyzed transcripts, and 3 team members (RR, SY, and CK) audited the data analysis findings.

### Ethical Considerations

The University of Ottawa Research Ethics Board (H01-16-02) granted approval for the study. All participants provided written informed consent before their interview; no personal information was recorded.

## Results

### Participant Characteristics

Table 1 summarizes the sample and participant characteristics. All participants’ practices were located in an urban setting in Ontario. Participants’ experience in using an EMR system ranged from 3 to 15 years. Overall, 5 of the participants were part of a group practice using the family health organization’s capitated payment model, 3 of the participants were from a family health team (FHT) practice model, and 1 participant was from an independent practice. In addition, 5 of the participants identified themselves as the information technology (IT) leader in their clinic. A total of 4 participants used the EMR system PS Suite, 3 used IndiviCare, and 1 worked with Nightingale On Demand.

**Figure 1:**
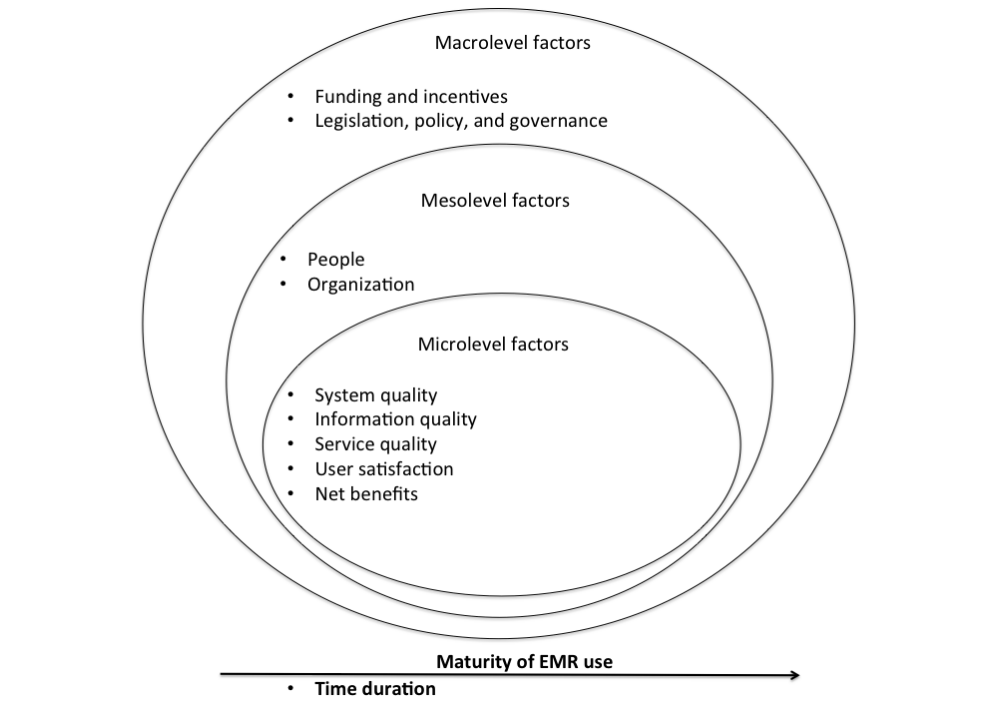
Dimensions emerging from the data. EMR: electronic medical record.

Patterns from the data were categorized into themes. In this study, themes refer to barriers and facilitating factors that influenced participants’ use of advanced EMR features. A total of 10 themes emerged from the data: 9 themes directly mapped to the dimensions of the CAF and one new theme was derived from our analysis. The dimensions from the framework that directly mapped to the 9 themes were system quality; information quality; service quality; user satisfaction; net benefits; people; organization; legislation, policy, and governance; and funding and incentives. Figure 1 shows the dimensions from the CAF that emerged from the data and the one new dimension (maturity of EMR use) that was derived from our analysis.

**Table 1:** Respondents’ characteristics.

Participants	Age range (years)	Sex	Primary care model	Experience using electronic medical records (years)	Information technology lead
P1	51-60	Male	FHT^a^	15	Yes
P2	61-70	Female	Independent practice	3	No
P3	61-70	Male	FHT	10	Yes
P4	41-50	Female	FHO^b^	7	Yes
P5	30-40	Male	FHO	7	Yes
P6	51-60	Male	FHO	15	Yes
P7	30-40	Female	FHT	4	No
P8	41-50	Male	FHO	4	No
P9	61-70	Female	FHO	9	No

^a^FHT: family health team.

^b^FHO: family health organization.

### Theme 1: System Performance (Microlevel)

The CAF defines the dimension, *system quality*, as the reliability of the system’s performance, features, and security and is estimated in terms of performance and reliability, based on the system’s response times for standardized tasks, integration with workflow, user-friendliness, and security [16].

Several participants explained that the quick response time for standardized tasks was a system performance factor that facilitated their use of advanced EMR features:

When I receive an abnormal test result I get it right away and I don’t need to wait for the next day.

P2, age 61-70 years

However, 2 participants mentioned that the drug database feature was not user friendly. Owing to the limitations of this feature, participants used mobile or Web-based drug database applications that were not part of the EMR software as they had an easier interface and quicker response time:

It’s so confusing…but I can write the same thing in my app…it’s just easier to read and it’s quicker.

P4, age 41-50 years

Participants also described system reliability as a barrier to using advanced EMR features (eg, EMR feature not working).

### Theme 2: Completeness of Information (Microlevel)

The dimension *information quality* is defined as the completeness and accuracy of information in addition to the timeliness and relevance of information [16]. Another facilitating factor is the completeness and relevance of information provided by the EMR drug database feature:

The system is more sophisticated than the last time…it will show me the various dosage forms…that are available.

P1, age 51-60 years

A few participants were concerned about the completeness and relevance of information provided by the EMR graph feature. These limited their ability to plot and view the trend of a patient’s test results:

It’s a terrible graph…because it’s not temporally organized…so it’s useless as a graph. (P3, age 61-70 years)

### Theme 3: User Training and Technical Support (Microlevel)

The dimension *service quality* is defined as user training and ongoing technical support and availability of support [16]. Participants were asked if they had an IT specialist on-site to support the EMR system. A total of 6 participants raised the issue of vendors’ insufficient ongoing technical support to enhance clinic performance, limited ongoing training for advanced EMR use, and ineffective user training. Technical support was not available on-site unless it was paid out of pocket or if a staff member communicated with the vendor.

### Theme 4: Perceived Usefulness of Electronic Medical Record Features, Perceived Impact on Productivity, and Perceived Impact on Quality of Care (Microlevel)

The CAF cites *user satisfaction* as one category that measures the dimension *satisfaction*, defined as the subjective opinions of users with regard to their perceived expectations; value; information, system, and service quality; and use of the system. Lau et al [16] assessed the framework’s user satisfaction component using indicators of perceived usefulness and value of the system, perceived impact on productivity and integration with workflow, and perceived impact on quality of care [16].

According to several participants, certain EMR features (eg, recall system and diabetic flow sheets) were useful and improved their quality of care, for example:

If there’s a drug recall, you can find all the patients who are on that drug and call…them to come in. So it’s amazing what you could do which you couldn’t do on a paper chart.

P4, age 41-50 years

Overall, 2 participants stated that using the EMR feature to assess cardiovascular risk was time consuming and inefficient, thus impacting productivity and preventing them from using this advanced ready-made feature. One participant described the use of the cardiovascular risk feature as challenging, in that it was not fully integrated into their EMR system, necessitating the use of other online tools to calculate risk:

Anything that’s inefficient is dangerous because it creates a barrier for people to do it. It promotes transcription errors. You move the data manually, you’re going to type a key wrong.

P6, age 51-60 years

### Theme 5: Change in Provider Efficiency, Net Cost, and Care Quality (Microlevel)

The CAF portrays *net benefits* as quality, access, and productivity. The framework assesses quality using indicators such as changes in provider effectiveness and appropriateness of care, whereas productivity is measured by indicators of change in provider efficiency, such as the time needed to assess a patient and clinician workflow [16]. The framework also refers to productivity as the change in net costs in terms of cost savings [16].

Participants reported improved workflow efficiency and improved patient efficiency when certain advanced EMR features were used. One participant described how workflow efficiency and patient efficiency were enhanced when they used a customized referral letter template to expedite a specialist referral: “So when I see an abnormal result I can send a referral at that time and its more efficient for me” [P2, age 61-70 years].

Overall, 2 participants suggested that change in productivity was a barrier to their use of advanced EMR features because of the additional cost associated with the EMR system, particularly maintaining, supporting, and upgrading the system to ensure effectiveness and efficiency. Other associated costs included after-sales support from vendors and hiring additional staff to deal with paper documents that were not electronically deposited into the EMR:

Since the EMR, we had to hire one person whose job was just to scan stuff in before the e-fax came.…I’m paying someone a full-time job just to scan, which is out of my pocket, which is created because of this technology.

P4, age 41-50 years

Furthermore, the quality of provider effectiveness and appropriateness of care were adversely affected when participants could not access patients’ test results from hospitals, in the EMR system. Participants mentioned wasting time searching for unavailable laboratory results instead of using that time for other tasks.

### Theme 6: Roles and Personal Characteristics (Mesolevel)

The CAF defines the dimension *people* as the individuals or groups involved, their personal characteristics and expectations, and their roles and responsibilities vis-à-vis the HIS [17].

The framework uses an individual’s age, gender, experience, and position (eg, being an IT leader) to measure personal characteristics and roles [17]. One participant with over 10 years of EMR experience, who was also the IT leader, described how they exploited the system:

I am too far into using EMRs.…I just do what EMR permits.…I really exploit the system.

P1, age 51-60 years

On the contrary, another participant (P2) with 3 years of experience using an EMR system revealed that they train their patients to remember when to do blood tests rather than use the reminder feature to prompt the physician for patient preventive services. Clearly, the participants’ characteristics and roles impacted their use of advanced EMR features.

### Theme 7: Return on Value and Infrastructure (Mesolevel)

The CAF categorizes *organization* as how the HIS fits with the organization’s strategy, culture, and structure or processes, as well as information, infrastructure, and return on value [17]. The framework defines return on value of HIS adoption in terms of cost benefit and effectiveness. Infrastructure is measured in terms of technical architectures, level of integration, and the privacy or security in place or planned [17].

Only a few participants stated that the return on value of advanced EMR features was a barrier to the use of these features. One participant said that the electronic fax feature was expensive and not reliable, so their clinic continued to use a paper-based process:

And that’s a problem with the software. They have an Internet faxing version, but they charge a fortune for it…and it has problems with capacity and reliability.

P6, age 51-60 years

Most participants noted that their inability to directly transfer documents among the EMR system and hospitals and pharmacies was a barrier. The majority of participants reported that they received laboratory results directly into their EMR system from private laboratories. However, most hospital results are faxed, scanned, and added to the patient’s chart, which was another barrier. The OLIS feature facilitates searching for missing laboratory results. However, some participants mentioned that not all hospital laboratory results were available in OLIS. If they were, the amount of paper that clinics received from hospitals would decrease:

If I go to [the patient’s] chart, I will see if their lab results are actually available through the EMR’s access to OLIS….If I can do that, then I don’t need all that printed paper.

P3, age 61-70 years

### Theme 8: Governance and Privacy Laws (Macrolevel)

Some participants were concerned about the lack of leadership in addressing poor EMR infrastructure, namely, lack of direct links with hospitals and pharmacies. According to one participant:

The fact that we can’t get stuff from hospital…There’s no technical problem. There’s no leadership that puts together the infrastructure and secures it to do it the way it’s supposed to be done. That’s all we’re missing, leadership…the government can fix two things. One, they could tell the people who supply the software whom they certify, that they have to provide turnkey end-to-end service. And number two, the government actually can help create the connectivity between us and the pharmacies, us and the hospitals.

P6, age 51-60 years

Furthermore, 2 participants were concerned about the security and privacy of patient charts because of legislation allowing the Ontario government to access patient data.

### Theme 9: Funding (Macrolevel)

A total of 2 other participants noted that they did not receive enough government funding to cover all the EMR system expenses. As one participant said:

[The program] didn’t cover everything but it was great, but then they stopped that…then this ongoing and maintaining, it’s all out of our pockets.

P4, age 41-50 years

### Theme 10: Maturity of Electronic Medical Record Use

Participants were directly asked how their use of EMRs for performing clinical tasks had evolved since adoption. The CAF does not have a category to account for the different maturity stages of the user, so a new category was developed. The CAF describes factors that impact the success of EMR adoption at a moment in time, whereas the new theme describes how these factors evolve over time.

Overall, 2 participants stated that their use of EMRs for performing clinical tasks had not evolved effectively since adoption. They noted flaws such as technical errors with the laboratory requisition feature; poor feature design for prescribing medication doses; and excessive scanner use because of the inability to electronically transfer documents among the EMR and some hospitals and pharmacies, which was needed to support continuity of care over time. Such flaws limited these participants from using the system to its maximum capacity. As one participant explained:

There’s way too much paper handling. Why is a person sitting at a scanner all day long? Why are we still waiting?

P6, age 51-60 years

However, most participants agreed that their use of the EMR system to perform clinical tasks had improved since its adoption. Several participants revealed the importance of using certain advanced EMR features (eg, electronic fax and Hospital Report Manager) to facilitate patient care delivery and reduce paper work. As one participant said:

We get features that now allow us to run almost a paperless office that did not exist when we first started

P5, age 30-40 years

As such, the use of advanced features to facilitate patient care delivery and reduce paper work demonstrates that these physicians’ use of the EMR system is maturing as they are able to incorporate advanced EMR features into their workflow.

Furthermore, using the electronic fax and Hospital Report Manager is considered advanced EMR use as physicians have incorporated these features into their clinical process as a way to facilitate CDPM. These features allow physicians to electronically access patient’s results and limit the need to scan paper documents into the EMR, thereby reducing the wait time of physicians accessing patient’s results. Thus, these features can improve patient care by decreasing the wait time during an appointment as the physician searches for the patient’s results or the possibility of human error when scanning paper documents into the EMR, such as support staff mismatching scanned results to a patient’s chart.

Theme 10 shows the need to have a temporal dimension to EMR evaluation to see what types of emerging issues will arise over time. The CAF looks at a more generic set of adoption factors, whereas theme 10 highlights the need to identify specific factors that facilitate EMR use that will emerge over time.

## Discussion

This study explores primary care physicians’ use of EMR systems to support CDPM. Most participants highlighted factors that facilitated their use of advanced EMR features. However, participants continue to experience barriers.

### Principal Findings and Comparison With Prior Work

#### Microlevel Factors

Most participants mentioned that system quality and information quality factors, such as quick response time for standardized tasks (eg, receiving blood test results), and the feature’s provision of complete and relevant information facilitated their use of advanced EMR features. However, participants reported unreliability as a barrier (eg, EMR feature not working), and a few participants also found the drug database feature to be non–user friendly.

Studies have recommended involving users in system design to address such technical factors [2,31,33]. As suggested in one study, professional associations, such as OntarioMD, could influence vendors by imposing standards and publishing specifications so that EMR features would be designed to benefit physicians [5].

Several participants noted that insufficient technical support and inadequate user training on the part of the vendor was a barrier. In addition, lack of on-site technical support from the vendor created additional costs such as hiring staff to address technical issues. A program such as QIDSS [13] could help address this barrier by helping physicians make better use of EMR data to improve clinical performance.

User satisfaction emerged from the data in terms of participants’ perceived usefulness of an EMR feature as well as its perceived impact on both productivity and quality of care. Although several participants noted that EMR features (eg, recall system and diabetic flow sheets) supported their quality of patient care, for others, certain EMR features (eg, data entry and cardiovascular risk feature) were inefficient and time consuming, thus a barrier to their productivity.

A systematic review recommended discussing the usefulness of a given EMR feature, demonstrating its ease of use, and having fellow physicians demonstrate the feature [34]. OntarioMD’s Peer Leader program is a network of clinicians with several years of EMR experience. These individuals support practices in Ontario to advance their EMR use [35]. Such a program can help address the user satisfaction barriers identified in our study.

#### Mesolevel Factors

According to our findings, participants who were IT leaders and had more EMR experience were more likely than others to exploit the EMR system. These findings are consistent with the diffusion of innovations theory, which describes how characteristics of potential adopters (eg, expertise and perception of innovation) influence the success of innovation adoption [10]. Furthermore, a commonly cited infrastructure barrier was the inability to directly transfer documents among the EMR system and hospitals and pharmacies. This barrier has also been identified in other studies [5,36].

#### Macrolevel Factors

Lack of leadership in addressing poor interoperability among EMR systems and hospitals and pharmacies is an important macrolevel factor discussed by a few participants. A grounded theory study conducted in Ontario also noted the lack of connectivity among clinical EMRs and hospital laboratories [5]. The study recommended that OntarioMD could influence software development via standards and publishing future requirements and through financial support to improve the interoperability among EMR systems and other health care entities [5].

Legislation and funding also emerged as issues in the data. Some participants were uneasy regarding the security and privacy of patient charts because of legislation that allows the Ontario government to access patient data. Other studies have also shown that concerns about privacy and security of patient data are a barrier to EMR use because of the potential legal problems [34,37,38].

In addition, participants who were not part of an FHT practice felt that government funding was not sufficient to cover EMR expenses. These findings confirm those of other studies in which barriers related to insufficient funding influenced the adoption and use of EMRs [2,5,39].

#### Maturity of Electronic Medical Record Use

Most participants thought that their use of EMR systems had improved since adoption with the support of advanced EMR features (eg, electronic fax and Hospital Report Manager). Studies that assessed clinicians’ use of EMR systems found that longer EMR use led to improved outcomes (eg, greater expertise and improved patient care) [14,15]. Some of the key factors explored in this study could be measured over time to assess the different maturity stages of physicians’ use of advanced EMR features.

Key factors such as reliability, functionality, and user-friendliness of the EMR feature; technical support and user training; user satisfaction; productivity; return on value; and infrastructure could be assessed as part of the mature use of an EMR system either quantitatively using surveys or qualitatively through interviews. One possible method would be ranking the progress of each key factor for each advanced feature and the progress of mature use of these advance features. For example, for the advanced feature OLIS, its reliability, functionality, and user-friendliness could be ranked using a Likert scale that ranges from 0 to 5, where 0 indicates that the user strongly disagrees that OLIS is reliable, functional, and user friendly. Similarly, the progress of mature use can be assessed using a 5-point Likert scale, where 0 shows that the user strongly disagrees that the feature is fully integrated within their clinical workflow (eg, feature is not being used) and 5 implies that the user strongly agrees that the feature is fully integrated within their clinical workflow (eg, feature is used to access patient’s current and past test results to enable treatment decisions and, if applicable, results are shared with the patient at the point of care). A longitudinal analysis of a clinic would need to be done to measure the progress of these key factors over time and the progress of mature use of these advanced EMR features. Thus, the maturity of EMR use dimension extends the CAF by incorporating postadoption factors perceived by physicians to influence their use of advanced features and the effects of these factors over time to reflect the different maturity stages of the user.

An application of this extended CAF would be to evaluate the progress of advanced EMR feature use among primary care physicians. Another would be for physicians to identify potential factors within their practice that influence their use of advanced EMR features in reaching maturity and to make recommendations for improvements.

Furthermore, the extended CAF could be used by key stakeholders, such as Canada Health Infoway and OntarioMD, to assess the progress of advanced EMR feature use to inform future policies designed to sustain the momentum of advanced EMR feature use.

### Limitations and Strengths

One limitation of our study is the composition of the participant sample. OntarioMD assisted with recruiting participants by reaching out only to its peer leaders. Peer leaders are typically super users who could be biased favorably toward EMRs. Another limitation is that no participants were located in a rural setting. This group might report other barriers or motives. Researcher bias because of using directed content analysis is another limitation, as researchers are likely to find evidence supportive of their theory. Finally, participants might have answered questions a certain way to please the researcher [24]. Doing an audit trail minimized biased results.

In addition, as the type of EMR software investigated was dependent on the software used by participants, the study only involved 3 types of EMR software: PS Suite, IndiviCare, and Nightingale On Demand. This may have prevented us from observing other advanced EMR features available in other EMR software. Moreover, the EMR software we investigated were all OntarioMD certified, which provided additional benefits (eg, access to Hospital Report Manager, OLIS, and EMR funding eligibility). Other factors might have emerged had we investigated non–OntarioMD-certified EMR systems.

A key strength of this study is that physicians were interviewed in person, providing a deeper understanding of their responses and allowing them to demonstrate certain EMR features. This, in turn, allowed us to observe the barriers and facilitating factors experienced by participants. In addition, the credibility of this study was enhanced by coauthors auditing the results and 2 team members independently analyzing transcripts.

### Conclusions

In this study, 9 primary care physicians in Ontario discussed barriers and facilitating factors that influenced their use of advanced EMR features. This study also extended the CAF through the emergence of a new dimension regarding the maturity of users’ EMR use. The extended CAF can be used to support key stakeholders in tracking the use of advanced EMR features, which would support future policies. A future research direction could be the development tools (eg, survey or interview guide) to formally evaluate the extended CAF. Overall, our findings show that although primary care physicians’ use of EMR systems has improved, barriers remain and need to be addressed to further enhance the physicians’ use of advanced EMR features to facilitate CDPM.

## Appendix

Multimedia Appendix 1 Electronic medical record maturity model.

Multimedia Appendix 2 Clinical adoption framework.

Multimedia Appendix 3 Interview guide.

## Abbreviations

CAF: Clinical Adoption Framework
CDPM: chronic disease prevention and management
EMR: electronic medical record
FHO: family health organization
FHT: family health team
HIS: health information system
IT: information technology
OLIS: Ontario Laboratories Information System
QIDSS: quality improvement decision support specialist
