# Supplementary material for: Primary Care Physicians’ Experience Using Advanced Electronic Medical Record Features to Support Chronic Disease Prevention and Management: Qualitative Study
Source: JMIR Med Inform. 2019 Nov 29;7(4):e13318. doi: 10.2196/13318 (PMC6911232; doi:10.2196/13318)
Supplement: Multimedia Appendix 1 [file medinform_v7i4e13318_app1.docx]

| Level | Criteria | Capabilities |
| --- | --- | --- |
| 5 |  |  |
|  | Integrate | Use of portals, hubs; attachment to provincial e-health platforms sharing data from the EMR. |
| 4 |  |  |
|  | Population data use | Dashboarding of whole populations, acting upon the whole, performing population analysis at the practice level. |
| 3 |  |  |
|  | Look ahead/predict | Reminders and alerts are used at the point of care. Searches are done regularly and scheduled for review. |
| 2 |  |  |
|  | Early data use | Acting upon the output of episodic searches, quick entry tools, forms, calculators, etc. |
| 1 |  |  |
|  | Enter data | Documentation occurs electronically. Progress notes, forms, and other documents are entered into the EMR. |
| 0 |  |  |
|  | Paper | Processes are primarily paper-based. |
