# Supplementary figures and images for: Primary Care Physicians’ Experience Using Advanced Electronic Medical Record Features to Support Chronic Disease Prevention and Management: Qualitative Study
Source: JMIR Med Inform. 2019 Nov 29;7(4):e13318. doi: 10.2196/13318 (PMC6911232; doi:10.2196/13318)

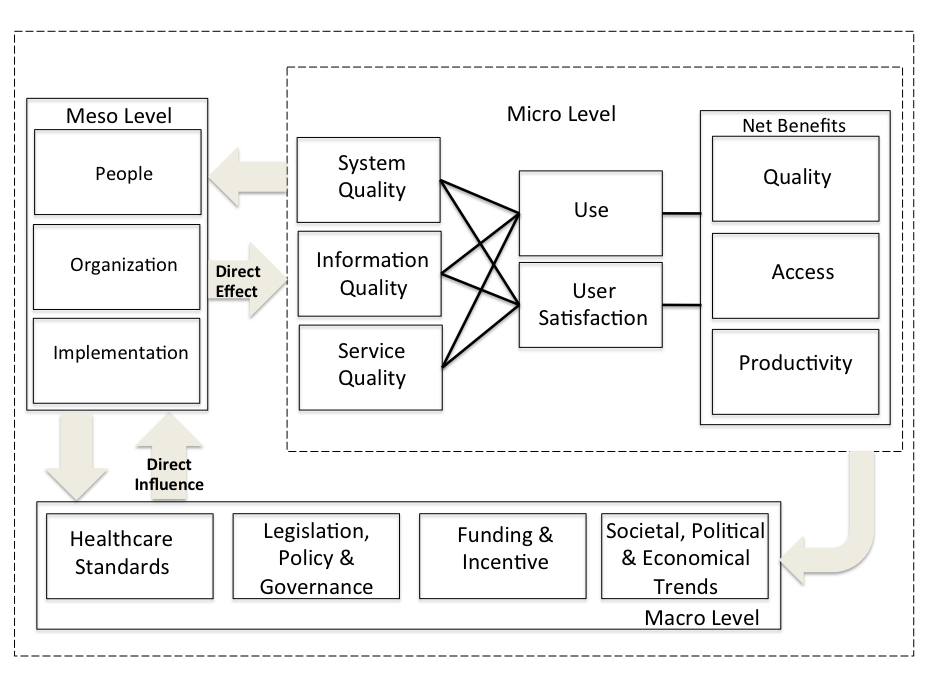

Supplement: Multimedia Appendix 2 [file medinform_v7i4e13318_app2.png]
