# Supplementary material for: Primary Care Physicians’ Experience Using Advanced Electronic Medical Record Features to Support Chronic Disease Prevention and Management: Qualitative Study
Source: JMIR Med Inform. 2019 Nov 29;7(4):e13318. doi: 10.2196/13318 (PMC6911232; doi:10.2196/13318)
Supplement: Multimedia Appendix 3 [file medinform_v7i4e13318_app3.docx]

| **Interview Guide** | | | |
| --- | --- | --- | --- |
| **Interview questions** | **Notes of participant’s answers** | | **Interviewer’s guide to answers** |
| 1. Could you tell me what type of model is this practice? |  | | Traditional Fee For Service  Family Health Group/Comprehensive Care Model  Family Health Network/Family Health Organization AND NOT a Family Health Team  Family Health Network/Family Health Organization AND a Family Health Team  Health Centre (Community Health Centre/Aboriginal Health Centre)  Nurse Practitioner Led Clinic  Other |
| 1. How long have you been using an EMR? |  | |  |
| 1. Could you tell me what age category you are in? |  | | A) Under 30; B) 30 – 40, C) 41 – 50, D) 51 – 60, E) 61 – 70, or F) 71 and older |
| **A. Before the physician sees the patient** | | | |
| 1. How does the nurse notify the MD the patient is ready and the room the patient is located? |  | | EMR feature (ex: In-office messaging)  Face-to-face  Whiteboard  Chart in door  Other |
| **B. When the physician sees the patient** | | | |
| 1. Does the MD review the patient’s chart prior to entering the patient’s room? | |  | [yes, no]  If no, when is the patient’s chart reviewed and where?  If yes, where does the MD review the patient’s chart?  At the office  At the nurses station  Other |
| 1. What information from the patient’s chart does the MD review? | |  | Lab tests  Preventive/screening services that are due  Problem list  Medication list  Clinical notes  Can you explain any EMR features used? |
| 1. What information does the MD enter into the patient chart when examining the patient? | |  | Current blood pressure  Plot/graph of the patient’s history of blood pressure or a lab test  Current weight  Medical history  Family history  Where is this information documented? In the EMR? Which features are used? What type? Are customized templates used? |
| 1. How do you know a patient is due or overdue for a test? | |  | e.g., cancer screening, mammograms, Pap tests, blood tests, blood pressure/cholesterol measurement, vitamin supplements, etc.  Who are the people or objects involved in this process?  What EMR features are used? (ex: In-office messaging) |
| 1. How do you diagnosis patients for chronic diseases? | |  | Who are the people or objects involved in this process?  Any EMR features used to help make the diagnosis? (e.g., plotting graphs of patient’s history glucose/blood pressure/weight level)? |
| **C. Patient Visit: Prescriptions** | | | |
| 1. How are in-office prescriptions written? | |  | Prescription pads in room  Prescription pads carried by doctor  EMR/printer (with an option to select drug and dosage from a list)  EMR and prescription sent directly to pharmacy (e-prescribing)  If EMR features are used, please describe in detail which features and the processes. |
| 1. Does the MD have a method to check for interactions/contraindications for medications? How does the MD do this? | |  | Are EMR features used?  Please describe in detail which features are used and the processes.  Is a mobile app used? If yes, what type and how is it used? |
| 1. How are prescriptions sent to the pharmacy? | |  | Faxed  Phone call  Prescription printed and given to patient to pick up at pharmacy  Prescription sent directly to pharmacy from EMR  Are EMR features used? Which features?  If prescriptions are directly sent to pharmacies from EMR, do you know how these pharmacies are linked with the EMR system at your clinic? (policies/protocols used?; Is it an in-clinic pharmacy?) |
| 1. Describe the process when a patient asks for a refill during an office visit? | |  | Who are the people or objects involved in this process?  What are the inputs and outputs?  What EMR features are used? |
| **D. Patient Visit: Lab tests** | | | |
| 1. How are laboratory tests ordered? | |  | Who are the people or objects involved in this process?  What are the inputs and outputs?  What EMR features are used? |
| 1. How are lab results returned to the clinic? | |  | Who gets them?  What do they do with them?  How are they matched with the patient’s chart?  Who matches them?  Any EMR features used? |
| 1. Do lab results go to the patient? | |  | How? Letter, phone call, email  Any EMR features used? |
| 1. Does the laboratory or hospital receive the orders directly from your EMR system? | |  | If so, how are these laboratories/hospitals linked with the EMR system at your clinic? How is this process done? Do you know what policies/protocols are being used?  If not, how does the laboratory or hospital receive the orders? |
| **E. Patient Visit: Referrals** | | | |
| 1. Describe the ways patients obtain referrals? | |  | Nurse/staff makes a call  MD fills out form  MD uses EMR and prints form to give to patient  Any EMR features used? |
| 1. How do you receive results of the referral? How do results get into the patient’s chart? | |  | Any EMR features used? |
| **F. End of patient’s visit** | | | |
| 1. When/where do you make your patient encounter notes (progress notes)? | |  | In clinic  In exam room  Out of exam room  At home, remote connect |
| 1. How are progress notes documented? | |  | Are EMR features used, which type of features? |
| 1. How is billing handled? Please describe the process, the people involved. | |  | Who documents the billings?  Are EMR features used, which type of features? |
| ***Wrap Up:*** | | | |
| 1. What do you like or dislike about your current EMR system when using it for CDPM? | |  | Please provide examples. |
| 1. Could you tell me how your use with the EMR for performing clinical tasks such as prescribing medication or ordering laboratory results has evolved from implementation to today? | |  | (To account for the process of maturity factor) |
| 1. Do you have an IT specialist that supports the EMR system at your practice? | |  | IT specialists such as Quality Improvement Data Support Specialists (QIDSS) |
| 1. Is the IT specialist funded by the government or by another source? | |  |  |
| 1. Have you been using the same EMR software since implementation or have you or will you be switching to another version? | |  |  |
| ***In Conclusion…*** | | | |
| 1. Do you have other comments or concerns about the use of EMRs for the prevention and management of patients with chronic diseases? | |  | Anything else you want to say/comment on? |
